# Central TSH Dysregulation in a Patient with Familial Non-Autoimmune Autosomal Dominant Hyperthyroidism Due to a Novel Thyroid-Stimulating Hormone Receptor Disease-Causing Variant

**DOI:** 10.3390/medicina57030196

**Published:** 2021-02-25

**Authors:** Jasna Suput Omladic, Maja Pajek, Urh Groselj, Katarina Trebusak Podkrajsek, Magdalena Avbelj Stefanija, Mojca Zerjav Tansek, Primoz Kotnik, Tadej Battelino, Darja Smigoc Schweiger

**Affiliations:** 1 Department of Endocrinology, Diabetes and Metabolic Diseases, University Children’s Hospital, University Medical Centre Ljubljana, Bohoričeva 20, 1000 Ljubljana, Slovenia; jasna.suputomladic@kclj.si (J.S.O.); urh.groselj@kclj.si (U.G.); magdalena.avbelj@mf.uni-lj.si (M.A.S.); mojca.zerjav-tansek@mf.uni-lj.si (M.Z.T.); primoz.kotnik@mf.uni-lj.si (P.K.); tadej.battelino@mf.uni-lj.si (T.B.); 2 Faculty of Medicine, University of Ljubljana, Vrazov trg 2, 1000 Ljubljana, Slovenia; katarina.trebusakpodkrajsek@mf.uni-lj.si; 3 Department of Paediatric Surgery and Intensive Care, University Medical Centre Ljubljana, Bohoričeva 20, 1000 Ljubljana, Slovenia; pajek.maja@gmail.com; 4 Clinical Institute for Special Laboratory Diagnostics, University Children’s Hospital, University Medical Centre Ljubljana, Vrazov trg 1, 1000 Ljubljana, Slovenia

**Keywords:** familial non-autoimmune autosomal dominant hyperthyroidism, FNAH, TSHR, radioiodine ablation therapy, central hypothyroidism

## Abstract

*Background and Objectives*. Familial non-autoimmune autosomal dominant hyperthyroidism (FNAH) is a rare cause of childhood hyperthyroidism. It is caused by the thyroid-stimulating hormone receptor (*TSHR*) gene variants. So far, only around 40 families with FNAH have been reported. Patients with activating *TSHR* variants demonstrated the same classical signs and symptoms of hyperthyroidism as seen in patients with Graves’ disease. Since 2012, ablative therapy is recommended to avoid relapses of hyperthyroidism and its consequences. *Case Presentation*. We presented a young adult male patient with a novel heterozygous *TSHR* disease-causing variant p.Arg418Lys (c.1253G>A) in the exon 10, who presented with a mild but progressive FNAH, with a follow-up since infancy. *Discussion*. Constantly suppressed TSH, including during the euthyreosis in childhood and hypothyreosis after iodine ablation therapy, suggested central dysregulation of the TSH secretion.

## 1. Introduction

The most common cause of hyperthyroidism in childhood is Graves’ disease [1]. A special entity is the congenital hyperthyroidism, which appears in infants due to trans placental transfer of maternally derived thyroid-stimulating immunoglobulin (TSI) antibodies [2,3,4,5]. Resistance to thyroid hormones (RTH) due to dominant pathogenic variants in *THRB* gene, a syndrome of reduced end-organ responsiveness to thyroid hormone (TH), is another rare cause of neonatal hyperthyroidism [6]. Less frequent causes for childhood hyperthyroidism are familial non-autoimmune autosomal dominant hyperthyroidism (FNAH) and sporadic congenital non-autoimmune hyperthyroidism (SCNAH), resulting from thyroid-stimulating hormone receptor (*TSHR*) gene variants [2,3,7]. *TSHR* gene is located on chromosome 14q31 and belongs to the glycoprotein hormone receptors and in a subfamily of the G-protein coupled receptors (GPCRs) [7,8,9,10,11]. An activating variant in the *TSHR* gene leads to a continuous expression of Gs/adenylyl cyclase pathway affecting the thyroid’s gland function and size, and the constant expression of Gq/11 phospholipase pathway, which is vital for the thyroid growth and thyroid hormones synthesis [9,12,13].

FNAH and SCNAH are characterized by persistent hyperthyroidism; typically, TSI (thyroid stimulating immunoglobulin) antibodies are absent and the relapse rate is high after thyrostatic medication and partial thyroidectomy. The only successful therapy for FNAH and SCNAH is a total thyroidectomy or radioiodine treatment. 

Patients with activating *TSHR* variants demonstrated the same classical signs and symptoms of hyperthyroidism as seen in patients with Graves’ disease. These include weight loss, hyperactivity, and tachycardia. Nonetheless, subclinical hyperthyroidism among NAH cases has been described in the literature [14]. Important clinical feature for FNAH is that persistent hyperthyroidism is present in at least two or more generations [2,15,16]. Another essential difference between these two entities is that SCNAH usually occurs in younger children and has a more severe course [2,7]. According to available data, there are 29 different *TSHR* disease-causing variants in families with FNAH and 20 different *TSHR* disease-causing variants in individuals with SCNAH [17], with a very little overlap of *TSHR* variants [2].

We present a long-term follow-up of a patient with mild but progressive FNAH due to a novel *TSHR* disease-causing variant.

## 2. Case Report

The proband was born at 40 weeks of gestation as a second child of unrelated parents; his birth length and weight were normal. There were no complications during pregnancy, birth, or the postnatal period. During pregnancy and childbirth, the then 31-years old mother was treated with methimazole due to hyperthyroidism. She was treated with methimazole since the age of 18 years. She did not complain of specific hyperthyroid symptoms and was not diagnosed until after her 3 years younger brother presented with hypertension and goiter. She presented with overt hyperthyroidism (thyroid-stimulating hormone (TSH) 0.03 mE/L, free thyroxine (T4) 35.8 pmol/L, and free triiodothyronine (T3) 9.4 pmol/L) before delivery. Three months after the delivery, her condition deteriorated with TSH 0.02 mE/L and free T4 42.2 and free T3 13.1 pmol/L. Since there was no improvement with anti-thyroid drugs, she received radioactive iodine 370 MBq 131-I. Despite partial ablation, during the 23 years of observation after radioiodine therapy, she never had another episode of hyperthyroidism. In euthyrotic state, her TSH remained suppressed. Furthermore, 7 years after radioiodine therapy, while she was for 6 years off any therapy, she was found hypothyrotic with inadequate TSH, indicating central hypothyroidism (TSH 3.592 mE/L, free T4 10.4 pmol/L (normal range: 11.5–22.7 pmol/L), and free T3 3.91 pmol/L (normal range: 3.5–6.5 pmol/L), and was since then permanently taking levothyroxine supplementation. Maternal uncle was treated with total thyroidectomy at the age of 20 years. Maternal grandmother was diagnosed with hyperthyroidism after her children at the age of 50 years and had no clear clinical signs of hyperthyroidism. However, she had a partial thyroidectomy due to goiter at a younger age. After a course of treatment with methimazole she subsequently received radioiodine therapy. All affected family members needed taking a thyroid hormone substitution after radioactive iodine. The proband’s brother was healthy.

**Figure 1:**
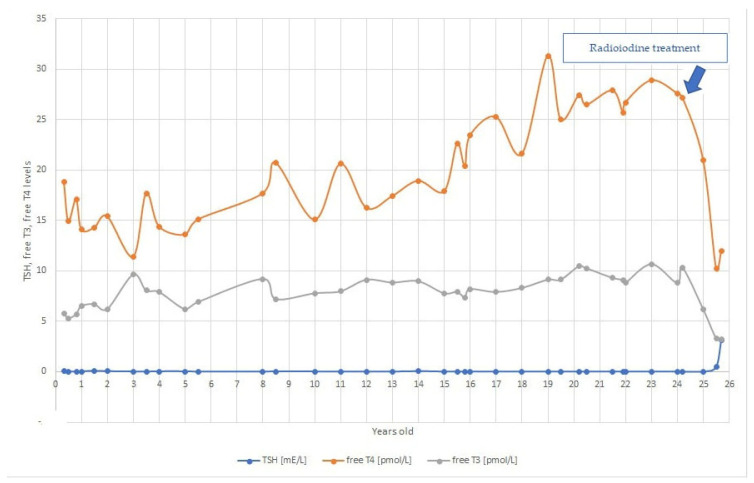
Thyroid hormone levels through the years of follow-up to iodine ablation therapy. Red represents free T4 levels, green, free T3 levels, and blue, TSH levels.

The proband was referred to our clinic as a 4 months old boy due to a family history of at that point unspecified thyroid disorder. He presented with normal thyroid hormones—free T4 18.8 pmol/L (normal range: 11.7–2.5 pmol/L) and free T3 5.8 (3.79–6.05 pmol/L) and suppressed TSH 0.07 mE/L (0.59–4.23 mE/L) (see Figure 1). Anti-Tg and anti-TPO antibodies were negative. The boy was followed at our outpatient clinic regularly. At the age of 11 months, he underwent a TRH stimulation test in evaluation of low TSH levels, while he had normal free T3 and free T4 levels. Blunted TSH response (peak TSH at 30 min was 0.79 mU/L) was observed after stimulation with TRH (see Table 1). Multiple thyroid ultrasounds were performed showing a normal thyroid with normal consistency and no thyroid nodules. He was clinically euthyroid with no psychomotor retardation. His growth was slightly accelerated (75th height velocity percentile). Thyroid hormone levels were in the euthyroid range with low TSH throughout childhood.

**Figure 2:**
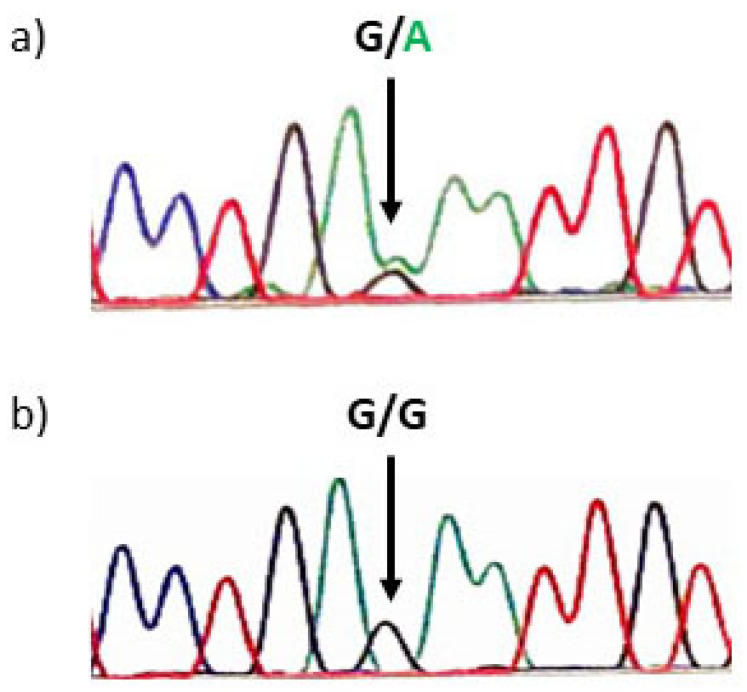
Novel *TSHR* gene variant NP_000360.2: p.Arg418Lys (NM_000369.2: c.1253G>A) in heterozygous state (**a**) and normal sequence (**b**).

**Figure 3:**
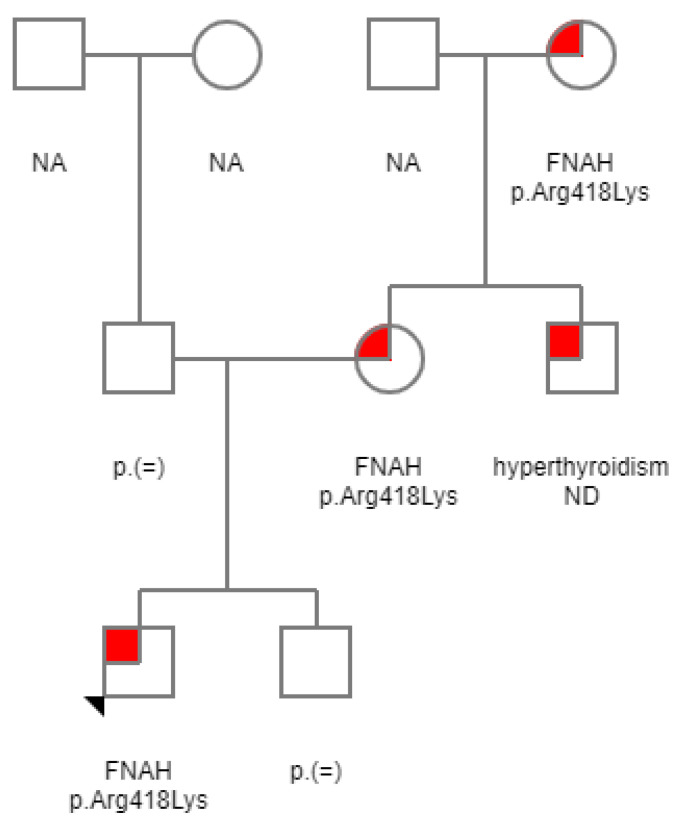
Family pedigree: family members with familial non-autoimmune autosomal dominant hyperthyroidism (FNAH) are indicated by red square; index patient is indicated by arrow. ND, not done; NA, not applicable.

At the age of 15 years, marginally increased systolic blood pressure was observed, diastolic blood pressure was normal, leading to a conclusion that this could be due to hyperthyroidism. His thyroid hormone values were free T4 22.62 pmol/L and free T3 7.93 pmol/L, and TSH was suppressed at 0.005 mE/L (see Figure 1). TSI antibodies were not detected. He had a tall stature (99th percentile (193 cm)) and normal BMI (22.3 kg/m^2^). After obtaining written informed consent, DNA analysis was carried out, and a heterozygous *TSHR* variant NP_000360.2: p.Arg418Lys (NM_000369.2: c.1253G>A) in the exon 10, was revealed (see Figure 2). This variant was not previously reported in patients with FNAH nor in the general population (gnomAD), in silico prediction tools predicted it to be causative (Mutation Taster: deleterious (1); Sift: damaging (0); CADD: score 28.8). Segregation analysis revealed that his mother and grandmother, both patients with hyperthyroidism, carried the same heterozygous variant (see Figure 3). Therefore, the detected variant was regarded as likely pathogenic according to the guidelines recommended by the American College of Medical Genetics and Genomics [18] with following grades: PM2: extremely low frequency in gnomAD population databases; PP1: co-segregation with disease in multiple affected family members in a gene definitively known to cause the disease; PP3: computational prediction tools unanimously support a deleterious effect on the gene; PP4: patient’s phenotype or family history is highly specific for a disease with a single genetic etiology. 

At the age of 16 years, he was treated at the cardiology department due to tachycardia with exertion. The heart ultrasound showed structurally and functionally normal heart with adequate aerobic capacity. Treatment was not started since he was otherwise asymptomatic at that time.

At the age of 20 years, his thyroid hormone level gradually increased and he was briefly commenced on thyrostatic therapy with methimazole 20 mg daily after the episode of weight loss and abdominal pain. No decline in thyroid hormone levels was observed after 1 month. Since the symptoms disappeared spontaneously, and he was not compliant to the treatment, methimazole was stopped. He reported no symptoms of hyperthyroidism, and there was no progression to overt hyperthyroidism in the next 3 years of follow-up. Physical examination showed mild tremor and sweating with a normal resting pulse and BMI (24 kg/m^2^). The thyroid gland volume was normal on clinical and ultrasound examination. However, at the cardiologic re-evaluation, his heart rate rose to 192 heartbeats per minute with ventricular extrasystoles during the cycle-ergometer exercise test. Echocardiogram showed no abnormalities and no signs of hypertension were found during 24-h ambulatory blood pressure monitoring. Treatment with radioiodine or total thyroidectomy was recommended. He agreed to radioiodine treatment. At the age of 25 years, he received 740 MBq 131-I with no complications. At the next appointment, lower thyroid hormone levels were observed—free T4 21 pmol/L, free T3 6.21 pmol/L, and TSH 0.01 mE/L. Four months after the treatment, he developed hypothyroidism with inadequate TSH level (free T4 10 pmol/L, free T3 3.3 pmol/L, and TSH 0.47 mE/L) and started treatment with levothyroxine (see Figure 1). He noticed some weight gain (2 kg) but otherwise reported no change in thyroid-related symptoms. Six months after radioiodine treatment while still hypothyroid despite taking 25 ug of levothyroxine, his TSH level was higher, yet inadequate, indicative of central hypothyroidism (TSH 3.10 mE/L, free T3 3.2 pmol/L (normal range: 4.1–6.7 pmol/L), and free T4 12.0 pmol/L (normal range: 13.4–21.3 pmol/L).

## 3. Discussion

FNAH is a rare form of non-autoimmune hyperthyroidism. Its prevalence is likely to be underestimated. The predominance of cases was diagnosed in Europe, and there is a slight female predominance [19]. Since the last update from TSH Receptor Mutation Database, 41 families with FNAH have been published [20]. We present a long-term follow-up of a young male with a mild FNAH and a novel *TSHR* disease-causing variant. According to the InterPro database [21], the variant is located at the extracellular border of the first transmembrane portion of TSHR. Another variant affecting the transmembrane domain was described in another family with FNAH in 2001 and was the first identified activating *TSHR* gene variant in a family with nonautoimmune hyperthyroidism [22]. The clinical presentation in our case was relatively mild initially, suppressed TSH being the only sign in childhood since infancy. In adolescence, subclinical hyperthyroidism progressed into overt hyperthyroidism and hyperthyroidism-related complaints emerged. The patient’s mother presented in adulthood with overt hyperthyroidism that could not be managed with medication and proceeded to iodine ablation. Hence her presentation, at least in adulthood, was more severe, but maternal grandmother had a milder course. Though specific genetics is associated with the severity of hyperthyroidism [2], intrafamilial phenotypic variability is observed in FNAH pedigrees. Previously described members of other families harboring *TSHR* germline variants show large differences in disease onset and the intensity of the hyperthyroidism [23]. Additional factors such as iodine intake and genetic background, may modify the phenotype [24].

European Thyroid Association (ETA) guidelines propose that all familial thyrotoxicosis cases with absence of evidence of autoimmunity should be evaluated for FNAH and if they display a *TSHR* germline variant, all other family members including asymptomatic and euthyroid family members should also be analyzed [25]. 

As FNAH is a rare condition, there are no randomized controlled trials that would provide information about the most successful line of treatment. Most of the information about the course of the disease is gathered from well-characterized case series. In FNAH, incomplete ablation or antithyroid drugs resulted in frequent relapses with the known pediatric complications of hyperthyroidism [26]. Long-term treatment with antithyroid drugs can control hyperthyroidism in some cases, however, further thyroid enlargement has been reported [27]. ETA guidelines strongly recommend the complete ablation of the thyroid tissue by total thyroidectomy followed by radioiodine administration [25]. 

Radioiodine therapy in our proband reduced the thyroid hormones below normal values and TSH remained inadequately low, indicating central dysregulation of TSH secretion. While the proband was observed only up to 6 months after the radioactive iodine therapy, in his mother, also treated for FNAH, inappropriate TSH response to hypothyroidism was observed 7 years after partial radioiodine ablation. In patients with activating *TSHR* variants, there is a limited amount of information on a long-term TSH response after treatment. An exceptional case, having inappropriate TSH response to severe hypothyroidism 14 years after radioiodine therapy, was reported and discussed by Jaesche et al. [28]. From previous reports, additional cases of inappropriate TSH response to hypothyroidism [4,29] and persistent suppression of TSH in euthyrotic state [17,30,31,32] can be deduced. However, there are also reports demonstrating appropriate TSH in relation to normal thyroid hormones or hypothyroidism [26,33]. We could summarize that prolonged TSH dysregulation occurs in a subset of patients with activating *TSHR* variants. The factors rendering the patients prone to such outcome remain elusive. It seems that our proband and his mother, carrying the same disease-causing variant at the extracellular border of the first transmembrane region of the *TSHR,* had up to the time of publication a similar clinical course, including central TSH dysregulation. Nevertheless, genetic variants reported in other patients with central TSH dysregulation affect distinct parts of the protein, including the first [4], third [29], and sixth [28] transmembrane domains and second extracellular loop [32]. Furthermore, adjacent to the variant Met626Ile, reported in the patient with well-founded long-term TSH suppression [28], are variants Ala627Val and Asp633Tyr associated with also well-founded normal central TSH response [26,33], making any conclusions based on specific genetic variants unreliable. 

A previously proposed mechanism behind TSH suppression after radioiodine treatment of hyperthyroidism was pituitary atrophy. Triiodothyronine has a strong suppressive effect on TSH levels. A lag time was noted in TSH recovery in patients with hyperthyroidism, and it was theorized that this lag corresponded to the time needed for atrophied thyrotrophes to regain function. Pituitary atrophy was confirmed in animal models and post-mortem analysis of human pituitary glands has indicated both gross atrophy and morphologic evidence of inactivity at the cellular level [34]. Nevertheless, a blunted TSH response to TRH stimulation in infancy and low TSH through all his youth in the presence of the euthyroid levels of free T3 and free T4 suggests another mechanism of TSH suppression at the pituitary level. We speculate that constant TSH suppression in our patient could be associated with constitutional activation of centrally located TSHR. It is shown that TSHR is expressed also outside the thyroid gland, including the hypothalamus, where it is suggested that a feedback mechanism is at play, modulating TRH and ultimately TSH secretion, preventing drastic swings in thyroid function [35] and pituitary, where TSH receptor RNA sequences are functionally expressed on the surfaces of folliculo-stellate cells [36]. Although thyroid hormones provide the pre-dominant feedback control, folliculo-stellate cells may act via paracrine mechanisms to fine-tune that response, avoiding drastic swings in TSH as thyroid function fluctuates [37]. Delayed recovery of the TSH responsiveness after radioactive iodine therapy is described also in some patients with euthyrotic Graves’ disease [38,39], where suppression of TSH is explained by antibody activation of pituitary TSHR, which regulates TSH secretion through ultrashort-loop feedback mechanism [35]. 

Further follow-up of thyroid function and TSH levels in our patient is needed, particularly, as only partial ablation was performed. 

To conclude, we presented a case with a mild but progressive FNAH, which emphasized the importance of the follow-up of patients with asymptomatic or subclinical FNAH. Our proband experienced a significant decrease in thyroid hormones after iodine ablation therapy, yet TSH remained inadequately low for up to 6 months after radioiodine treatment, as was all his life since infancy, which suggested thyroxin independent TSH regulation, possibly through centrally expressed mutated TSHR. Unexpectedly and persistently low TSH in euthyroid subjects could be taken as a possible sign of FNAH. A novel variant, which segregated with the phenotype in our pedigree, broadened the spectrum of *TSHR* activating variants.

## Figures and Tables

**Table 1:** TRH test results. TSH—thyroid stimulating hormone, T3—triiodothyronine, T4—thyroxine, ND—not determined.

Time (Minutes)	0	30	120
TSH (mE/L)	0.03	0.79	ND
Free T3 (pmol/L)	6.5	ND	8.0
Free T4 (pmol/L)	14.1	ND	16.4

## Data Availability

No new data were created or analyzed in this study. Data sharing is not applicable to this article.
